# A Novel Polyvalent Bacteriophage vB_EcoM_swi3 Infects Pathogenic *Escherichia coli* and *Salmonella enteritidis*

**DOI:** 10.3389/fmicb.2021.649673

**Published:** 2021-07-14

**Authors:** Bingrui Sui, Lili Han, Huiying Ren, Wenhua Liu, Can Zhang

**Affiliations:** College of Veterinary Medicine, Qingdao Agricultural University, Qingdao, China

**Keywords:** bacteriophage vB_EcoM_swi3, *Escherichia coli*, *Salmonella enteritidis*, biological characteristics, genomic analysis, phage therapy

## Abstract

A novel virulent bacteriophage vB_EcoM_swi3 (swi3), isolated from swine feces, lyzed 9% (6/65) of *Escherichia coli* and isolates 54% (39/72) of *Salmonella enteritidis* isolates, which were all clinically pathogenic multidrug-resistant strains. Morphological observation showed that phage swi3 belonged to the *Myoviridae* family with an icosahedral head (80 nm in diameter) and a contractile sheathed tail (120 nm in length). At the optimal multiplicity of infection of 1, the one-step growth analysis of swi3 showed a 25-min latent period with a burst size of 25-plaque-forming units (PFU)/infected cell. Phage swi3 remained stable both at pH 6.0–8.0 and at less than 50°C for at least 1 h. Genomic sequencing and bioinformatics analysis based on genomic sequences and the terminase large subunit showed that phage swi3 was a novel member that was most closely related to *Salmonella* phages and belonged to the *Rosemountvirus* genus. Phage swi3 harbored a 52-kb double-stranded DNA genome with 46.02% GC content. Seventy-two potential open reading frames were identified and annotated, only 15 of which had been assigned to functional genes. No gene associated with pathogenicity and virulence was identified. The effects of phage swi3 in treating pathologic *E. coli* infections *in vivo* were evaluated using a mouse model. The administration of a single intraperitoneal injection of swi3 (10^6^ PFU) at 2 h after challenge with the *E. coli* strain (serotype K88) (10^8^ colony-forming units) sufficiently protected all mice without toxic side effects. This finding highlighted that phage swi3 might be used as an effective antibacterial agent to prevent *E. coli* and *S. enteritidis* infection.

## Introduction

Pathogenic *Escherichia coli* and *Salmonella enteritidis* are the major opportunistic pathogens in animals and humans and are frequently reported worldwide (Majowicz et al., 2010; Yang et al., 2017; Jajere, 2019). Rearing animals on a large scale can increase pathogen infections. Particularly in young animals in high-density farming models, outbreaks caused by *E. coli* and *S. enteritidis* are usually associated with gastroenteritis and diarrhea and lead to serious infections and high mortality rates. To control enteric infections, antibiotics are widely used in breeding farms to treat infections. However, the widespread use of antibiotics had led to a series of problems, such as drug resistance, environmental pollution, and antibiotic residues in animal products (Paitan, 2018; Poirel et al., 2018; Jajere, 2019; Thames and Theradiyil Sukumaran, 2020). The prevention and control of bacterial infection urgently require the development of alternative antibiotic products.

Bacteriophages (phages) are viruses that exclusively infect bacteria and are widely distributed in the environment (Parikka et al., 2017). In the past decade, phages have demonstrated a promising alternative antibiotic role in the control of pathogenic bacteria in animals and humans (Chang et al., 2018; Rehman et al., 2019). Additionally, phages are a resource for many biotechnological applications, such as antimicrobial enzymes, bacteria typing, and phage display libraries (Salmond and Fineran, 2015). However, the host range of reported phages is commonly limited to a single species, and few phages can infect more than one species. Therefore, phage cocktails were developed to control polymicrobial infections in phage therapy. In our opinion, a broader-spectrum phage would presumably lead to fewer failures due to a mismatched host and phage combination. *Myoviridae* phages usually exhibit a broader host range than *Siphoviridae* and *Podoviridae* (Chibani-Chennoufi et al., 2004). Moreover, it is necessary to acquire a clear understanding of biology and genetic information to ensure effectiveness and safety before the use of phages. A suitable phage candidate for effective biocontrol should be a lytic phage with a broad host range against a variety of bacterial strains and should not carry virulence and pathogenicity genes in the genome. To date, the reported phages in the database have only been the tip of the iceberg, and most of them belong to the *Myoviridae* or the *Siphoviridae* families of tailed phages (Yu et al., 2006).

In this study, a novel polyvalent bacteriophage vB_EcoM_swi3 was isolated and characterized. Phage swi3 showed a wide host range; it could lyze 9% (6/65) of *E. coli* strain and 54% (39/72) of *S. enteritidis* strain, and it showed a good protective effect in a mouse model challenged with pathogenic *E. coli*. These data provide valuable information to assess the potential of phage swi3 as a biocontrol agent against pathogenic bacteria.

## Materials and Methods

### Bacterial Strains and Animals

Sixty-five pathogenic *E. coli* and 72 pathogenic *S. enteritidis* clinical isolates were used in this study (these strains were identified as different strains from different batches of diseased animals in Hebei, Shandong, and Jilin from 2010 to 2020, and drug sensitivity tests revealed different drug resistances). The bacterial strains were cultured in Luria–Bertani culture (LB) at 37°C and stored in 30% glycerol at −80°C.

Female BALB/c mice, 5 weeks of age (20–25 g in weight), were purchased from the Experimental Animal Center of Shandong, China.

All animal procedures were performed in strict accordance with the Regulations for the Administration of Affairs Concerning Experimental Animals, approved through the State Council of the People’s Republic of China (1988.11.1), and with the approval of the Animal Welfare and Research Ethics Committee at Qingdao Agricultural University.

### Propagation and Morphology of Phage Swi3

A pathogenic clinical strain of *E. coli* (serotype K88), hereafter named *E. coli* K88, was used in this study for phage isolation. Feces and seawater samples were collected from a pig farm in Shandong, China. Phage isolation was performed using the standard enrichment method as described before but with slight modifications (Lu et al., 2017). In brief, an overnight culture of 50 ml of *E. coli* K88 was cocultured with collected samples at 37°C for 24 h and then centrifuged at 12,000 rpm for 30 min. The supernatant was filtered with a sterile disposable membrane filter (0.22 μm). Then, 100 μl of the filtered supernatant was added to 100 μl of log-phase *E. coli* K88 and mixed with 3 ml of prewarmed NA top agar (0.7% agar), spread on an NA plate, and cultured for 4 h at 37°C. A single plaque was selected and picked from the plate. Then, the plaque was leached overnight with 0.9% normal saline. This procedure was repeated three times to obtain purified phages. Electron micrographs of purified phage particles were obtained according to a standard method (Kreienbaum et al., 2020). A 10-μl phage sample was dropped onto carbon-coated grids, negatively stained with 2% (w/v) aqueous uranyl acetate (pH 4.0) for 5 min, and air-dried. The samples were observed with a transmission electron microscope (HT7700, Hitachi, Japan) at 80-kV accelerating voltage.

### Host Range Determination of Phage Swi3

Sixty-five *E. coli* and 72 *S. enteritidis* clinical isolates were used to test the host range of phage swi3 by the spot method. Briefly, 10 μl of diluted phage suspension (1 × 10^5^ PFU/ml) was spotted on each bacterial lawn on agar plates and incubated at 37°C for 4 h. The presence of plaques was examined. Additionally, the efficiency of plating was tested as described before (Ding et al., 2020).

### Optimal Multiplicity of Infection of Phage Swi3

To determine the optimal multiplicity of infection (MOI) of phage swi3, *E. coli* K88 was cultured in LB broth at 37°C with shaking at 250 rpm until the early exponential phase (5 × 10^7^ CFU/ml). Phage swi3 was cocultured with *E. coli* K88 in 10 ml of LB broth at different MOIs (0.0001, 0.001, 0.01, 0.1, 1, and 10) (Abedon, 2016). A culture of bacteria without phage swi3 (0 MOI) was used as a control. After 2.5 h of incubation, the culture was centrifuged at 10,000 rpm for 10 min, and then the supernatant was used to determine the phage titers of phage swi3 by the double-layered agar method. The MOI resulting in the highest phage titer was considered an optimal MOI and used in subsequent large-scale phage production.

### One-Step Growth of Phage Swi3

The burst size and the latent period of phage swi3 were determined by one-step growth analysis as previously described (Sun et al., 2012). In brief, 200 μl of phage suspension (10^6^ PFU) was mixed with 200 ml of bacterial culture (5 × 10^6^ CFU/ml), incubated at 37°C for 5 min, and then centrifuged, and the cell pellet was resuspended in 500 μl of LB broth. Aliquots were taken, and the phage titers were immediately determined by the double-layered agar method. This assay was performed in triplicate.

### Thermal/pH Stability of Phage Swi3

The stability of phage swi3 was tested as previously described (Akhwale et al., 2019). Briefly, thermal stability was assessed by incubating the phage swi3 (4 × 10^8^ PFU/ml) at 40, 50, 60, 70, and 80°C, and aliquots (100 μl) were taken at 20, 40, and 60 min. To evaluate the stability of phage swi3 at various pH values (2, 3, 4, 5, 6, 7, 8, 9, 10, 11, 12, and 13), phage suspensions were incubated in LB broth with different pH values at 37°C for 1, 2, and 3 h and then assayed by the double-layered agar method. All assays were performed in triplicate.

### Sequencing and Genome Analysis of Phage Swi3

Phage genomic DNA was extracted using a Virus Genome Extract DNA/RNA Kit (Tiangen, Inc., Beijing, China) according to the manufacturer’s instructions. Then, the extracted genomic DNA was sent to Biozeron Company (Shanghai, China) for high-throughput sequencing on an Illumina HiSeq platform and assembled with ABySS, v2.0.2^1^. Contigs were assembled using the *de novo* assembly algorithm Newbler, version 3.0, with default parameters (Bao et al., 2014). Potential open reading frames were predicted and annotated using RAST^2^ and GeneMark^3^ (Besemer and Borodovsky, 2005; Aziz et al., 2008). Additionally, the genome sequence, the terminase large subunit, and the tail-associated protein amino acid sequence of phage swi3 were compared separately in the GenBank database of NCBI, and all homologous phages were selected to construct the phylogenetic tree of phage swi3 using the neighbor-joining method with default parameters in MEGAX software. The genomes of phage swi3, *Salmonella phage BP63* (KM366099.1), *Salmonella phage LSE7621* (MK568062.1), *Salmonella phage UPF_BP2* (NC_048649.1), and *Salmonella phage vB_SenM_PA13076* (MF740800.1) were compared and performed using Mauve software with a default parameter (Darling et al., 2004).

### Antibacterial Activity of Phage Swi3 in a Mouse Model Challenged With *E. coli* K88

To test the antibacterial activity of phage swi3, the 50% lethal dose (LD50) of *E. coli* K88 was first determined. In brief, 28 5-week-old female BALB/c mice were randomly divided into four groups. Each group was treated orally with different doses of *E. coli* K88 (10^6^, 10^7^, 10^8^, and 10^9^ CFU). The LD50 was determined based on the mortality of mice and used in subsequent challenge tests. To test the protective effect of phage swi3 on mice, 42 5-week-old female BALB/c mice were randomly divided into six groups (a–f). Groups a–d were inoculated orally with 10^8^ CFU of *E. coli* K88. Then, groups a–c were inoculated intraperitoneally with a single dose (10^5^, 10^6^, and 10^7^ PFU, respectively) of phage swi3 at 2 h after the bacterial challenge. Control group d was inoculated with an equivalent volume of LB instead of phage swi3, control group e was treated with 10^7^ PFU of phage swi3 without *E. coli* K88 challenge, and control group f only took an equal volume of LB orally. Then, the mice were observed daily for 5 days to record the clinical signs and cumulative mortality. At the same time, blood and feces were collected every 2 h on the first day and every 24 h after the first day to detect the metabolic dynamics of bacteria and phage swi3 in the mice.

### Nucleotide Sequence Accession Number

The sequence data of swi3 were deposited at GenBank under accession number MT768059.

## Results

### Morphology of Phage Swi3

**FIGURE 1:**
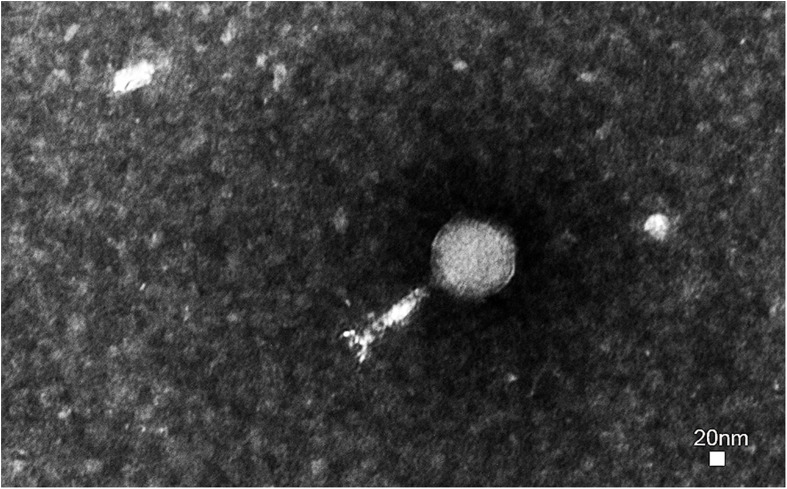
Morphology of phage swi3. Phage swi3 was negatively stained with 2% uranyl acetate and observed by transmission electron microscopy at an accelerating voltage of 80 kV. The scale bars represent 20 nm.

A novel virulent phage was isolated from *E. coli* K88 and observed by transmission electron microscopy. Phage swi3 has a regular icosahedral head (80 nm in diameter) and a contractile sheathed tail 120 nm in length (Figure 1). Thus, phage swi3 belonged to the family *Myoviridae*, order *Caudovirales*, following the current guidelines of the International Committee on Taxonomy of Viruses (ICTV). According to the novel universal system of bacteriophage naming, the suggested full name of phage swi3 will be vB_EcoM_swi3 (Lavigne et al., 2009).

### Host Range of Phage Swi3

The swi3 host range was determined using 65 *E. coli* strains and 72 *S. enteritidis* strains (Supplementary Table 1). Interestingly, phage swi3 showed characteristics of a wide range across species, not only having a certain lytic ability for six of the 65 *E. coli* strains but also having a lytic ability for 39 of the 72 *S. enteritidis* strains.

### The Growth of Phage Swi3

**FIGURE 2:**
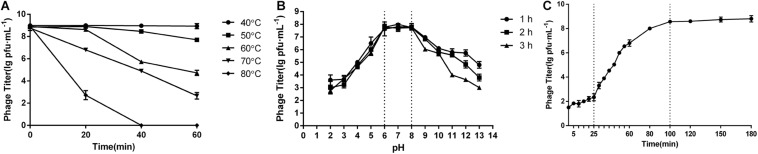
Biological characteristics of phage swi3. **(A)** One-step growth curve, **(B)** pH stability, and **(C)** thermal stability. Data are expressed as means. At the optimal multiplicity of infection of 1, the one-step growth analysis of swi3 showed a 25-min latent period with a burst size of 25-plaque-forming units (PFU)/infected cell. Phage swi3 remained stable both at pH 6.0–8.0 and less than 50°C for at least 1 h.

The biological characteristics of phage swi3 were measured by the double-layer plate method (Zhang et al., 2013). When the MOI was 1, phage swi3 had the highest titer of 6.4 × 10^8^ PFU/ml after proliferation. The one-step growth analysis showed that the incubation period of swi3 was approximately 25 min, after which there was a rapid increase in the number of released viral particles. It took about 75 min for swi3 to reach the growth plateau phase with a burst size of approximately 25 PFU/infected cells (Figure 2A). In addition, phage swi3 remained stable in the pH range of 6–8 (Figure 2B) and at a temperature less than 50°C for at least 1 h (Figure 2C).

### Genome Analysis of Phage Swi3

**FIGURE 3:**
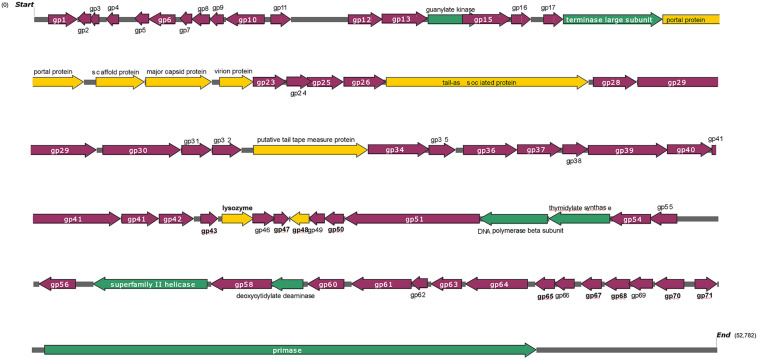
Genome structure of phage swi3. The arrow represents the direction of transcription. Different colors represent open reading frames with different predictive functions. The structural proteins are represented in yellow, blue indicates cleavage-related proteins, the proteins associated with transcriptional transcription are expressed in green, and hypothetical proteins are represented in purple.

The genome of phage swi3 was sequenced and analyzed. The general characteristics of the genome include a total of 52,782 bp with an overall GC content of 46.02%. Seventy-two *orf*s were predicted, 39 of which were positive-stranded, while the others were negative-stranded. Only 15 of 72 *orf*s were annotated as functional genes, including six structural-related genes (*orf* 19, *orf* 20, *orf* 21, *orf* 22, *orf* 27, and *orf* 33), seven genes associated with transcription and replication (*orf* 14, *orf* 18, *orf* 52, *orf* 53, *orf* 57, *orf* 59, and *orf* 72), and two lysis-related genes (*orf* 44 and *orf* 45). A detailed phage swi3 genome annotation showed that the *orf*s related to transcription and replication were mainly concentrated in the downstream part of the whole genome, while the structurally related *orfs* were mainly concentrated in the upstream part of the whole genome sequence (Figure 3).

### Phylogenetic Analysis of Phage Swi3

The phylogenetic tree of phage swi3 was constructed based on the genomic sequence, the terminase large subunit (encoded by *orf* 19) sequence, the tail-associated protein (encoded by *orf* 27), and the tail tape measure protein (encoded by *orf* 33), using the neighbor-joining method with the default parameters in MEGAX software.

Based on the terminase large subunit, most comparable phages had less than 60% homology, a total of 10 phages in NCBI showed high similarities (>94%), and all of them belonged to *Salmonella* phages. Only *Salmonella virus BP63* (KM366099.1) was included in ICTV, with 98% coverage rate and 97.73% identity. The phylogenetic tree showed that 11 phages belonged to two groups, phage swi3 and eight more phages belonged to the *Rosemountvirus* genus, and the other two phages belonged to the *Loughboroughvirus* genus (Supplementary Figure 1). Based on the whole-genome sequence, the phylogenetic tree was highly consistent with that of the terminase large subunit; all homologous phages were divided into two groups, and phage swi3 belonged to the *Rosemountvirus* genus. Additionally, all the homologous phages were *Salmonella* phages (Supplementary Figure 2). The genomic comparison results showed that no rearrangement or inversion occurred in the phage swi3 genome (Supplementary Figure 3).

Only two tail-related proteins were annotated among the known *orfs*: the tail-associated protein (encoded by *orf* 27) and the tail tape measure protein (encoded by *orf* 33). Their phylogenetic trees were constructed. Based on the tail-associated protein, only four comparable phages were found in the NCBI database with a high homology (>82.7%), and all of them were *Salmonella* phages. Other phages in the database showed less than 45% homology (Supplementary Figure 4). Based on the tail tape measurement protein, all homologous phages also belonged to *Salmonella* phages with more than 70% similarity (Supplementary Figure 5).

### Protective Effects of Phage Swi3 in a Mouse Model Challenged With *E. coli* K88

**FIGURE 4:**
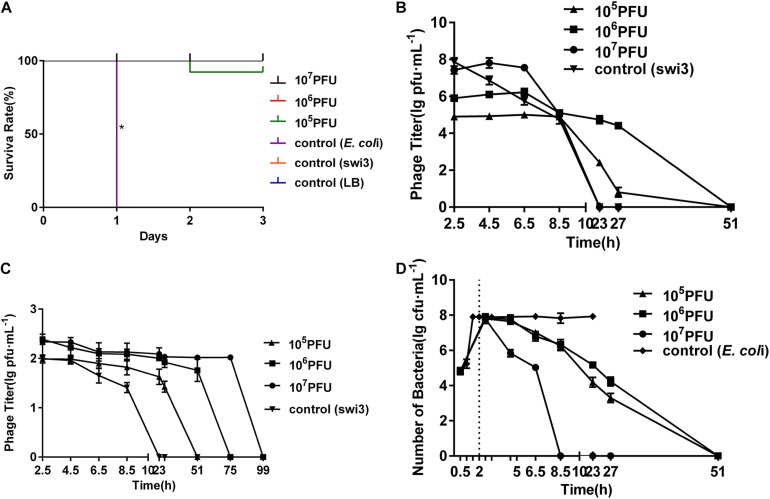
Phage swi3 rescued mice from *Escherichia coli* K88 infection. **(A)** Survival rates. The mice were inoculated orally with 10^8^ colony-forming units of the *E. coli* K88 strain. The data were expressed as means ± SD and statistically analyzed using the one-way ANOVA method at a level of significance *p < 0.05 (GraphPad Software, Inc., San Diego, CA, United States). At 2 h later, different phage doses were introduced intraperitoneally to treat the challenged mice. **(B)** Dynamic changes of bacteria in blood in different groups. **(C)** Dynamic changes of phage in blood in different groups. **(D)** Dynamic changes of phage in feces in different groups.

The LD50 of *E. coli* K88 on mice was determined as 10^8^ CFU, and the protective effects of phage swi3 were tested in a mouse model challenged with *E. coli* K88. There were obvious protective effects of phage swi3 in the bacteria-challenged mouse model (*p* < 0.05). After the challenge with *E. coli* K88, all mice in control group d died within 1 day; their lungs, liver, spleen, and kidneys showed varying degrees of bleeding and swelling, and *E. coli* K88 was isolated from these diseased organs. All mice in control groups f and e had good health until the end of the experiment, and no organ lesions were found (Supplementary Figure 6). In groups a–c, different doses of phage swi3 showed a good protective effect on *E. coli* K88-challenged mice. Compared with control group d, except for one mouse in group a that died on the second day, all mice survived, and no organ lesions were found (Figure 4A). The results indicated that a single intraperitoneal injection of swi3 (10^6^ PFU) at 2 h after oral challenge with *E. coli* K88 could sufficiently protect all mice without toxic side effects.

In addition, the dynamics of bacteria and phages in the blood and feces were measured. *E. coli* K88 entered the blood and reached its peak (10^8^ CFU/ml) 1.5 h after inoculation. Phage swi3 was intraperitoneally injected into the mice, detected in the blood in a short time, and then cleared *E. coli* K88 within 8.5–51 h in the blood; no bacteria or phage could be detected after 51 h (Figures 4B,C). Meanwhile, the dynamics of phage swi3 in feces were also measured. With the increase in phage inoculation dose, the phage detection time in feces was prolonged, ranging from 23 to 99 h (Figure 4D).

## Discussion

*Escherichia coli* and *Salmonella enteritidis* are becoming increasingly important opportunistic pathogens worldwide that endanger animal breeding industries. The difficulties in treating multidrug-resistant strains and the specificity of phage therapy prompted researchers to focus on phage therapy. In this study, we isolated a novel lytic phage swi3 from swine feces, which showed a broad host range against multidrug-resistant *E. coli* and *S. enteritidis*.

Generally, bacteriophages show strong host specificity and usually display a species-limited host range. To our knowledge, few phages infecting more than one species of bacteria have been reported (Park et al., 2012; Amarillas et al., 2016; Pham-Khanh et al., 2019). In this study, phage swi3 was isolated from *E. coli* k88, but the phylogenetic tree analysis results based on the whole-genome sequence and the terminase large subunit showed that all phages with homology in the database were *S. enteritidis* phages, and the tail-associated protein and the tail tape measure protein were also *S. enteritidis* phages, which attracted our attention. In general, the host range of phages is related to their genomes. Therefore, two tail-related proteins were selected for BLAST analysis with the sequences in the database. The phylogenetic tree based on the tail-associated protein and the tail tape measure protein also showed a homology with the *S. enteritidis* phages. Thus, we hypothesized that phage swi3 might be able to lyze *S. enteritidis* phages. To test our hypothesis, *S. enteritidis* strains were selected to detect the host range of phage swi3, and 39 of 72 strains could be lyzed. Therefore, the phage swi3 could lyze not only *E. coli* but also *S. enteritidis*. The verification test showed that the host bacterial species of the phage could be preliminarily inferred through homology analysis, which was helpful to determine the phage host spectrum. It was speculated that the interspecific recognition mechanism of phage swi3 might be related to its infection process. The phage swi3 belonged to *Myoviridae*, which first bound to the corresponding receptor on the surface of the host bacteria through the receptor binging protein (RBP) on the tail fiber so that the phage adsorbed to the surface of the host bacteria and then initiated the process of phage infection (Bertozzi Silva et al., 2016). The phage swi3 could lyze *Salmonella* and *E. coli*, so its RBP needed to recognize receptors not only on the surface of *E. coli* but also on the surface of *S. enteritidis*. In addition, it was possible that the unknown *orf*-encoded proteins of phage swi3 were involved in the host adsorption process, which requires further analysis and verification. Phage swi3 could infect *E. coli* and *S. enteritidis* efficiently and showed a wide host range, which made it suitable for use as a biological control agent.

In the past 10 years, the application of phages in the prevention and control of bacterial diseases in animal reproduction has been widely reported. The results of animal experiments *in vivo* are different, but they all showed that phage preparations have no toxicity or side effects on the animal body, and due to the characteristics of phage proliferation with the host bacteria, a low-dose phage preparation can have a good bactericidal effect (Schneider et al., 2018; Balasubramanian et al., 2019; Kaabi and Musafer, 2019). In this study, a mouse model challenged with *E. coli* K88 was used to test the protective effect of phage swi3. The results showed that a low dose of 10^6^ PFU of phage swi3 could protect mice from *E. coli* attack without any obvious side effects, and the phage was cleared within a short period of time because of the animal immune clearance response, which was consistent with previous reports (Li et al., 2015).

In conclusion, phage swi3 had a broad host spectrum including *E. coli* and *S. enteritidis*; it can clear bacteria in animals within a short time without side effects and has a potential value in the treatment of bacterial diseases.

## Data Availability Statement

The datasets presented in this study can be found in online repositories. The names of the repository/repositories and accession number(s) can be found below: https://www.ncbi.nlm.nih.gov/genbank/, MT768059.

## Ethics Statement

The animal study was reviewed and approved by the Animal Welfare and Research Ethics Committee at Qingdao Agriculture University.

## Author Contributions

BS performed the experiments, analyzed the data, and wrote this manuscript. LH, HR, and WL performed the experiments. CZ designed the experiments and revised the manuscript. All authors contributed to the article and approved the submitted version.

## Conflict of Interest

The authors declare that the research was conducted in the absence of any commercial or financial relationships that could be construed as a potential conflict of interest.

## Footnotes

1 http://www.bcgsc.ca/platform/bioinfo/software/abyss

2 http://rast.nmpdr.org

3 http://opal.biology.gatech.edu/GeneMark/
