# Supplementary material for: A Novel Polyvalent Bacteriophage vB_EcoM_swi3 Infects Pathogenic Escherichia coli and Salmonella enteritidis
Source: Front Microbiol. 2021 Jul 14;12:649673. doi: 10.3389/fmicb.2021.649673 (PMC8317433; doi:10.3389/fmicb.2021.649673)
Supplement: Supplementary file 1 [file Table_1.pdf]

Table S1

EOP of phage swi3 in 65 *E. coli* strains and 72 *S. enteritidis* strains

Strain name	Susceptibility results								EOP
	PEN	DOX	FFC	ENR	CIP	CTX	NEO	CRO	
E. coli 1	R	I	S	R	S	S	R	S	-
E. coli 2	R	R	S	I	S	I	R	S	-
E. coli 3	R	R	S	I	I	S	R	S	0.14
E. coli 4	R	R	I	S	S	I	R	S	-
E. coli 5	R	R	I	I	S	S	R	S	-
E. coli 6	R	R	I	I	I	I	R	S	-
E. coli 7	R	R	I	I	S	S	R	S	-
E. coli 8	R	R	R	R	S	S	I	S	-
E. coli 9	R	R	S	I	I	I	R	S	-
E. coli 10	R	R	S	I	S	I	I	S	-
E. coli 11	R	R	I	R	R	S	I	S	-
E. coli 12	R	R	I	I	I	I	I	I	-
E. coli 13	R	R	R	I	S	R	I	S	-
E. coli 14	R	R	R	I	I	R	I	R	-
E. coli 15	R	R	R	I	I	S	I	S	-
E. coli 16	R	R	R	R	I	S	R	S	-
E. coli 17	R	R	R	R	R	S	I	S	-
E. coli 18	R	R	R	I	S	S	R	S	-
E. coli 19	R	R	R	R	I	S	R	S	-
E. coli 20	R	R	S	S	S	S	S	S	-
E. coli 21	R	R	S	R	R	I	R	S	-
E. coli 22	R	R	R	R	S	I	R	S	-
E. coli 23	R	R	R	R	R	I	R	R	0.63
E. coli 24	R	R	R	R	R	I	I	I	-
E. coli 25	R	R	R	R	R	R	R	R	-
E. coli 26	R	R	I	S	S	I	I	S	-
E. coli 27	R	R	R	I	I	I	I	S	-
E. coli 28	R	R	I	I	R	I	I	I	-
E. coli 29	R	R	R	R	R	I	I	I	-
E. coli 30	R	R	R	I	I	S	I	S	-
E. coli 31	R	R	R	R	R	S	R	I	-
E. coli 32	R	R	R	I	I	S	I	S	-
E. coli 33	R	R	R	R	R	I	I	I	-
E. coli 34	R	R	R	S	S	S	R	S	-
E. coli 35	R	R	R	R	R	R	I	S	-
E. coli 36	R	R	R	R	I	I	R	S	-
E. coli 37	R	R	I	R	R	I	I	I	-
E. coli 38	R	R	S	I	I	S	R	S	-

E. coli 39	R	I	I	S	S	I	R	S	-
E. coli 40	R	R	I	I	S	S	I	R	-
E. coli 41	R	R	R	I	I	I	R	S	-
E. coli 42	R	R	I	I	S	S	R	S	18.75
E. coli 43	R	R	R	R	S	S	I	I	-
E. coli 44	R	R	S	I	I	I	R	S	-
E. coli 45	R	R	S	I	S	I	I	S	-
E. coli 46	R	R	I	R	R	S	I	S	1.25
E. coli 47	R	R	I	I	I	I	R	I	-
E. coli 48	R	R	R	R	S	R	I	S	-
E. coli 49	R	R	R	I	I	R	I	R	-
E. coli 50	R	R	R	I	I	S	R	S	-
E. coli 51	R	R	S	R	I	S	I	S	-
E. coli 52	R	R	R	R	R	R	R	S	-
E. coli 53	R	R	R	I	S	S	I	I	-
E. coli 54	R	I	S	R	I	S	R	S	-
E. coli 55	R	R	S	S	S	S	R	R	-
E. coli 56	R	R	S	R	R	I	S	S	-
E. coli 57	R	R	R	R	S	R	S	S	-
E. coli 58	R	R	R	R	R	I	R	R	-
E. coli 59	R	R	I	R	R	I	I	I	-
E. coli 60	R	I	R	R	S	R	R	I	-
E. coli 61	R	R	I	S	S	I	I	I	-
E. coli 62	R	R	R	I	I	S	I	S	-
E. coli 63	R	R	I	I	R	I	I	I	-
E. coli 64	R	R	R	R	R	I	I	I	0.12
E. coli K88	R	R	S	S	S	I	S	I	1
S. enteritidis 1	R	R	R	I	S	S	R	I	-
S. enteritidis 2	R	R	R	R	S	I	R	I	-
S. enteritidis 3	R	R	R	R	S	S	R	I	-
S. enteritidis 4	R	R	R	S	I	R	R	S	0.63
S. enteritidis 5	R	R	S	R	I	S	R	S	-
S. enteritidis 6	R	R	S	R	I	I	R	S	-
S. enteritidis 7	R	R	S	R	R	I	R	I	-
S. enteritidis 8	R	R	I	R	R	I	R	S	2.66
S. enteritidis 9	R	R	S	R	R	I	I	S	-
S. enteritidis 10	R	R	R	S	R	I	I	S	8.13
S. enteritidis 11	R	R	R	R	R	S	I	R	-
S. enteritidis 12	R	R	R	I	R	S	S	R	-
S. enteritidis 13	R	I	R	R	S	I	S	R	-
S. enteritidis 14	R	I	R	R	R	I	S	S	-
S. enteritidis 15	R	I	S	R	R	I	S	S	-
S. enteritidis 16	R	R	R	R	R	I	R	R	0.13
S. enteritidis 17	R	R	R	I	R	S	R	I	0.66

S. enteritidis 18	R	R	R	R	R	S	R	S	0.47
S. enteritidis 19	R	R	R	I	S	S	R	S	0.41
S. enteritidis 20	R	R	I	S	S	I	R	S	5.00
S. enteritidis 21	R	R	I	R	I	I	R	R	0.13
S. enteritidis 22	R	R	R	R	I	I	R	R	-
S. enteritidis 23	R	I	R	R	S	I	I	R	-
S. enteritidis 24	R	R	R	I	R	I	I	R	1.56
S. enteritidis 25	R	R	R	I	S	R	I	S	-
S. enteritidis 26	R	R	R	S	R	R	I	S	-
S. enteritidis 27	R	R	R	S	R	I	I	S	0.09
S. enteritidis 28	R	R	R	R	I	I	I	S	3.13
S. enteritidis 29	R	R	R	R	I	I	I	S	-
S. enteritidis 30	R	R	S	R	I	I	S	R	0.78
S. enteritidis 31	R	R	R	R	S	I	S	R	-
S. enteritidis 32	R	R	R	R	S	S	S	R	0.78
S. enteritidis 33	R	R	R	R	R	S	I	R	1.13
S. enteritidis 34	R	R	R	I	R	S	I	S	1.41
S. enteritidis 35	R	R	R	R	R	S	I	S	0.98
S. enteritidis 36	R	R	R	S	R	I	I	S	-
S. enteritidis 37	R	R	R	S	R	I	I	R	0.08
S. enteritidis 38	R	R	I	I	S	I	I	R	-
S. enteritidis 39	R	I	R	R	S	I	R	R	0.11
S. enteritidis 40	R	I	R	R	S	I	R	S	-
S. enteritidis 41	R	R	R	R	R	I	R	S	0.16
S. enteritidis 42	R	R	R	R	R	I	R	S	0.09
S. enteritidis 43	R	R	R	I	R	I	R	I	0.94
S. enteritidis 44	R	R	R	S	I	I	R	I	-
S. enteritidis 45	R	R	S	R	I	I	I	S	0.31
S. enteritidis 46	R	R	R	R	I	I	I	S	-
S. enteritidis 47	R	I	R	R	S	S	I	S	0.13
S. enteritidis 48	R	R	R	R	S	S	I	S	0.12
S. enteritidis 49	R	R	R	R	S	S	I	S	6.25
S. enteritidis 50	R	R	R	I	R	S	I	R	-
S. enteritidis 51	R	R	R	S	R	S	I	R	-
S. enteritidis 52	R	R	R	S	R	R	I	R	0.47
S. enteritidis 53	R	R	I	S	R	R	S	I	1.34
S. enteritidis 54	R	R	R	I	R	I	S	S	-
S. enteritidis 55	R	R	R	I	R	I	S	S	0.09
S. enteritidis 56	R	R	R	I	R	I	S	R	-
S. enteritidis 57	R	R	R	R	R	I	S	R	1.09
S. enteritidis 58	R	R	R	R	S	S	R	S	1.88
S. enteritidis 59	R	R	R	R	S	I	R	S	-
S. enteritidis 60	R	R	R	R	I	I	R	S	-
S. enteritidis 61	R	R	R	R	I	S	R	S	0.94

S. enteritidis 62	R	I	R	R	I	I	R	I	-
S. enteritidis 63	R	I	R	R	S	I	I	S	-
S. enteritidis 64	R	R	R	R	S	I	I	I	0.13
S. enteritidis 65	R	R	R	R	R	I	I	R	0.14
S. enteritidis 66	R	R	S	I	R	I	I	R	-
S. enteritidis 67	R	R	R	S	R	S	I	S	1.56
S. enteritidis 68	R	R	R	I	R	S	I	S	2.50
S. enteritidis 69	R	R	R	R	R	R	S	R	1.22
S. enteritidis 70	R	R	R	R	R	S	S	R	0.08
S. enteritidis 71	R	I	R	S	R	I	I	I	0.25
S. enteritidis 72	R	R	R	R	R	I	I	I	-

Note: PEN, Penicillin; DOX, Doxycycline hydrochloride; FFC, Florfenicol; ENR, Enrofloxacin; CIP, Ciprofloxacin; CTX, Cefotaxime; NEO, Neomycin; CRO, Ceftriaxone; R, resistance; I, intermediate; S, sensitive; -, no lysis.
