# Supplementary material for: A Novel Polyvalent Bacteriophage vB_EcoM_swi3 Infects Pathogenic Escherichia coli and Salmonella enteritidis
Source: Front Microbiol. 2021 Jul 14;12:649673. doi: 10.3389/fmicb.2021.649673 (PMC8317433; doi:10.3389/fmicb.2021.649673)
Supplement: Supplementary file 3 [file Data_Sheet_2.PDF]

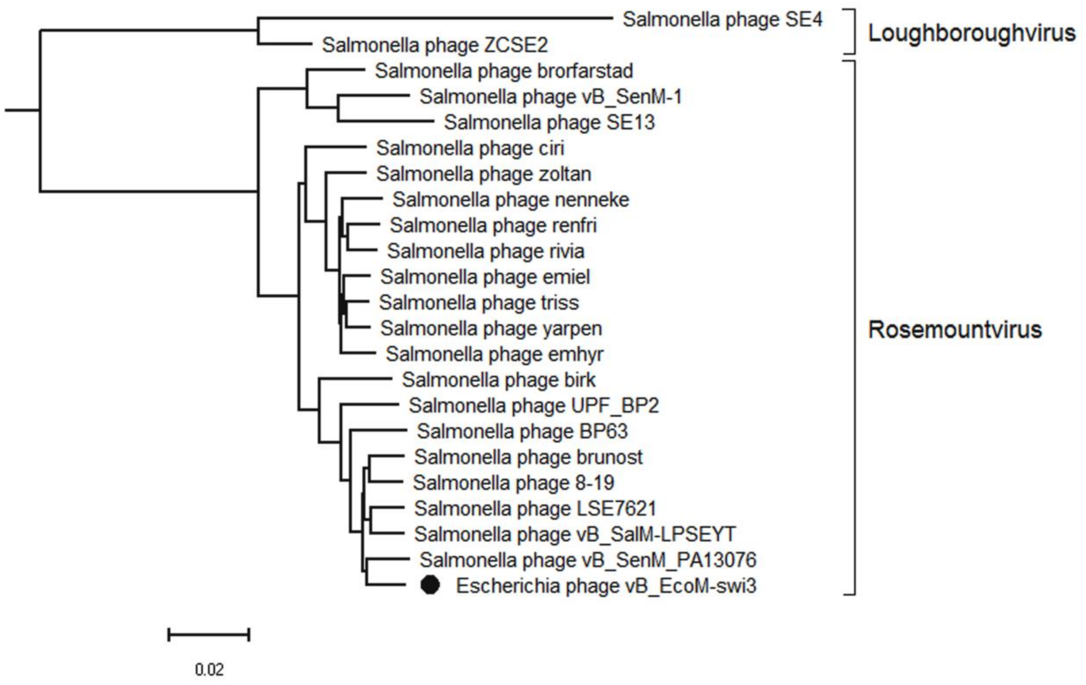

Fig. S2. The phylogenetic tree was constructed based on the whole-genome using the Neighbor-Joining method with default parameters in MEGA 7.0 ● represent phage swi3.
