# Supplementary material for: A Novel Polyvalent Bacteriophage vB_EcoM_swi3 Infects Pathogenic Escherichia coli and Salmonella enteritidis
Source: Front Microbiol. 2021 Jul 14;12:649673. doi: 10.3389/fmicb.2021.649673 (PMC8317433; doi:10.3389/fmicb.2021.649673)
Supplement: Supplementary file 4 [file Data_Sheet_3.PDF]

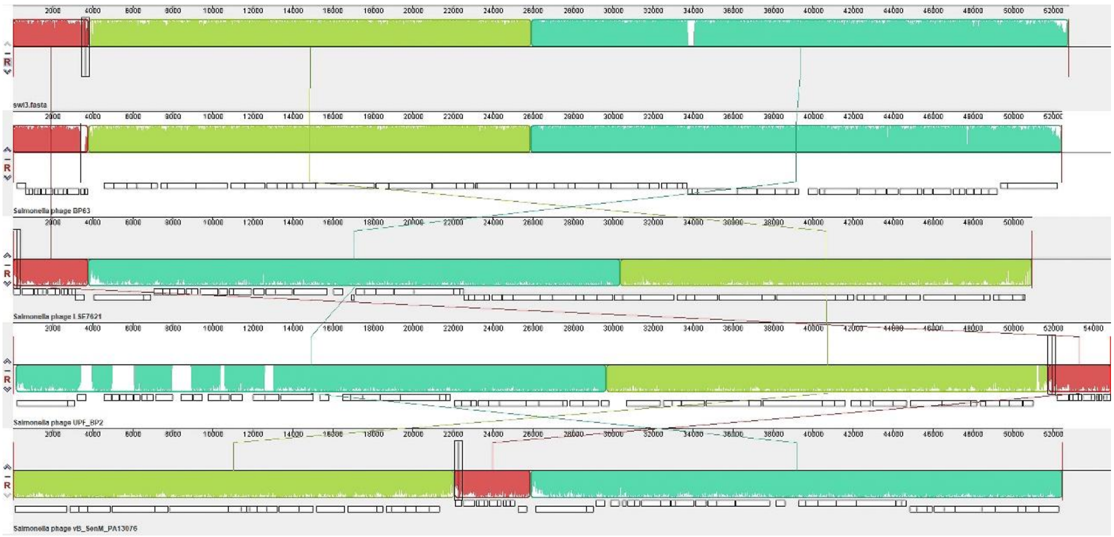

Fig. S3. Genomic comparation of phage swi3. There was no rearrangement or inversion among phage swi3, BP63 and LSE7621, but a genomic rearrangement was found in phage BP2 and PA13076.
