# Supplementary material for: A Novel Polyvalent Bacteriophage vB_EcoM_swi3 Infects Pathogenic Escherichia coli and Salmonella enteritidis
Source: Front Microbiol. 2021 Jul 14;12:649673. doi: 10.3389/fmicb.2021.649673 (PMC8317433; doi:10.3389/fmicb.2021.649673)
Supplement: Supplementary file 7 [file Data_Sheet_6.PDF]

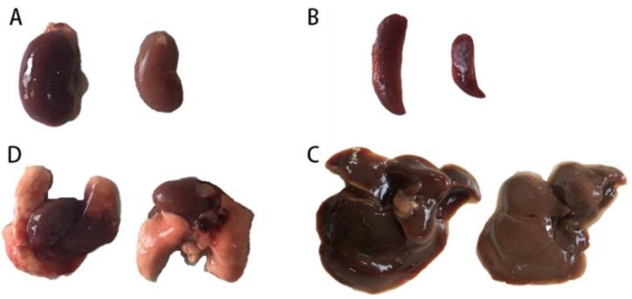

Fig. S6. The organs necropsy of mouse. The organs necropsy of challenged mouse (left) and control mouse (right). Compared with control organs, (A) kidneys and (B) spleens of challenged mouse were swelling and congestion, (C) lungs and (D) livers had bleeding.
